# The PyMVPA BIDS-App: a robust multivariate pattern analysis pipeline for fMRI data

**DOI:** 10.3389/fnins.2023.1233416

**Published:** 2023-08-24

**Authors:** Sajjad Torabian, Natalia Vélez, Vanessa Sochat, Yaroslav O. Halchenko, Emily D. Grossman

**Affiliations:** ^1^Visual Perception and Neuroimaging Lab, Department of Cognitive Sciences, University of California, Irvine, Irvine, CA, United States; ^2^Computational Cognitive Neuroscience Lab, Department of Psychology, Harvard University, Cambridge, MA, United States; ^3^Lawrence Livermore National Laboratory, Livermore, CA, United States; ^4^Department of Psychological and Brain Sciences, Dartmouth College, Hanover, NH, United States

**Keywords:** fMRI, MVPA, PyMVPA, BIDS, BIDS-App

## Abstract

With the advent of multivariate pattern analysis (MVPA) as an important analytic approach to fMRI, new insights into the functional organization of the brain have emerged. Several software packages have been developed to perform MVPA analysis, but deploying them comes with the cost of adjusting data to individual idiosyncrasies associated with each package. Here we describe PyMVPA BIDS-App, a fast and robust pipeline based on the data organization of the BIDS standard that performs multivariate analyses using powerful functionality of PyMVPA. The app runs flexibly with blocked and event-related fMRI experimental designs, is capable of performing classification as well as representational similarity analysis, and works both within regions of interest or on the whole brain through searchlights. In addition, the app accepts as input both volumetric and surface-based data. Inspections into the intermediate stages of the analyses are available and the readability of final results are facilitated through visualizations. The PyMVPA BIDS-App is designed to be accessible to novice users, while also offering more control to experts through command-line arguments in a highly reproducible environment.

## 1. Introduction

Machine learning and multivariate approaches to fMRI data analyses provide insights into the functional organization of the brain not possible with standard univariate analyses. Several software packages have emerged that facilitate these approaches, but adopting them often comes with the significant cost of writing scripts to connect each software package to the idiosyncratic data structures of the user's organization or lab.

The Brain Imaging Data Structure (BIDS) (Gorgolewski et al., 2016) was developed as a means to circumvent these up-front technical challenges. The standard describes an organization of files and specifications for file names, and outlines a uniformly structured metadata that has been widely adopted in the field. The BIDS structure has the additional advantages of being easily extended to different modalities of neurophysiological data [e.g., EEG (Pernet et al., 2019) and MEG (Niso et al., 2018) extensions]. This innovation has also facilitated the development of a growing number of BIDS-Apps, portable pipelines for common neurophysiological analyses and computational approaches. Thus, by adopting BIDS, the neuroimaging community can reap the benefits of a common data structure, encouraging easier usage of open-source pipelines and facilitating reproducibility.

Here we describe the PyMVPA BIDS-App, a pipeline developed to seamlessly integrate BIDS-compatible fMRI data with the powerful multivariate pattern analysis (MVPA) functionality of PyMVPA, an open-source package that makes use of machine learning tools in Python (Hanke et al., 2009). The app performs ROI-based as well as searchlight pattern analysis (Kriegeskorte et al., 2006), including classification with cross-validation performance estimation (Refaeilzadeh et al., 2009) and representational similarity analysis (RSA) (Kriegeskorte et al., 2008), both in volumetric space and on the surface. Statistical testing for classification analysis has also been implemented in the pipeline to enhance interpretability, specifically through permutation testing. By automating the entire multivariate analysis, the app obviates the need for coding to read event timing and label information, while enabling inspection into intermediate stages of the analyses, and creates visualization of patterns in NIfTI (volumetric) or GIfTI (surface-based) format. The PyMVPA BIDS-App also adds functionality to PyMVPA for improved beta-estimation in event-related designs using the least squares single (LSS) approach (Mumford et al., 2014). The app is deployed through a container image (Gorgolewski et al., 2017), which makes it possible to use with Docker or Singularity (Kurtzer et al., 2017) and allows users to deploy the app within high-performance computing (HPC) environments without a time-consuming installation process. The PyMVPA BIDS-App is designed to be accessible to novice users, while also offering experts advanced command-line arguments for more control in a highly reproducible environment.

## 2. The pipeline

### 2.1. BIDS specification

The PyMVPA BIDS-App forms a bridge between PyMVPA, a freely available tool for multivariate pattern analysis, and BIDS formatted datasets. It takes advantage of BIDS standard for organizing and describing neuroimaging and behavioral data, which specifies a directory organization, metadata, and naming scheme to promote accessibility in data sharing and neuroimaging tools. Importantly, this BIDS-App requires datasets to be BIDS compatible and also to have gone under pre-processing using fMRIPrep (Esteban et al., 2018), another BIDS-App that applies commonly implemented preprocessing steps and returns the functional data aligned to standardized template spaces. fMRIPrep outputs functional data in both NIfTI and GIfTI formats, which will be used by PyMVPA BIDS-App for volumetric and surface-based analysis, respectively.

**Figure 1:**
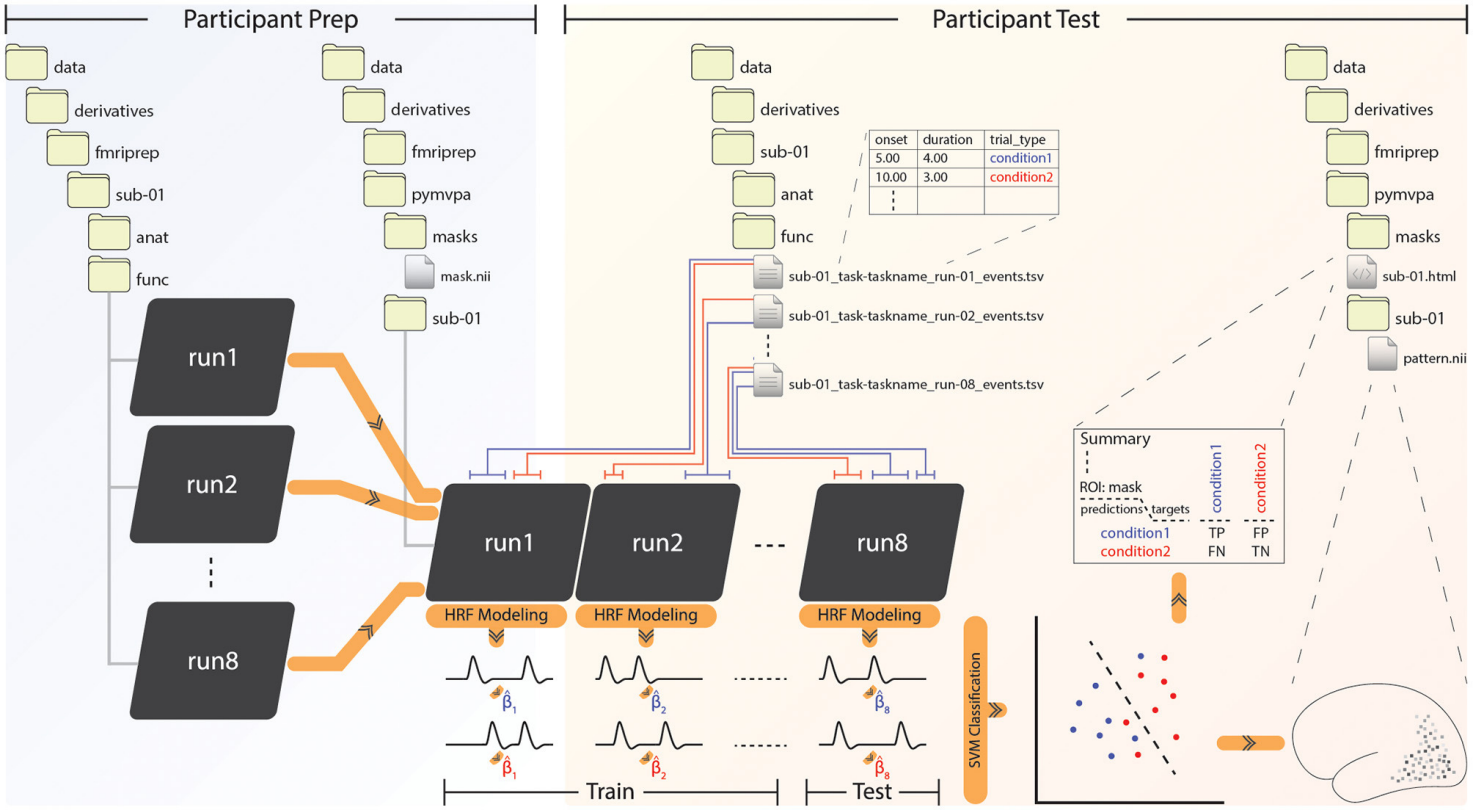
The PyMVPA BIDS-App pipeline. **Left**: Participant preparation level, receives input from fMRIPrep and concatenates all functional runs of each subject into a single file. In the case of a volumetric ROI-based analysis, the user must place a NIfTI (.nii) file specifying the region of interest (ROI) in the “masks” folder. **Right**: At the participant test level, PyMVPA classifies beta estimates given by the hemodynamic response function (HRF) modeling of experimental conditions, or finds similarities between the betas. Note that only classification of betas estimated through ordinary least squares (OLS) is depicted here, but the app is also capable of LSS beta estimation and computing representational similarity. The results are rendered through HTML reports together with visualized NIfTI or GIfTI patterns. The example here shows a volumetric NIfTI output file, which will also be gzip compressed and given in .gz format.

Users interact with the app through the command-line interface, as we demonstrate in section 3 with example invocations of both fMRIPrep and PyMVPA BIDS-App via Docker. The pipeline described below and detailed in Figure 1 shows the most common implementation of the PyMVPA BIDS-App. Optional deviations are noted at various points throughout the paper, which include running the pipeline in whole-brain searchlight mode or within regions of interest (ROI), and classification vs. representational similarity analysis. To maximize transparency, a complete and detailed list of the command-line arguments has been documented through GitHub (https://github.com/bids-apps/PyMVPA).

### 2.2. Concatenation and masking the functional scans

The PyMVPA BIDS-App prepares BIDS compatible individual participant images for classification and RSA by concatenating relevant functional scans, as depicted in Figure 1 under the participant prep phase. This process is performed for volumetric analyses using the fslmerge function from FSL suite of tools (Jenkinson et al., 2012), which provides a set of command-line utilities for the analysis of NIfTI images. The resulting merged NIfTI will be saved for each subject under its output folder. For surface-based analyses, concatenation of GIfTI files is performed later, at the beginning of the participant test phase.

The user has the option to restrict the analysis to an ROI as specified within a NIfTI (or GIfTI) mask file they place under a folder created by the app and named “masks” (see Figure 1). In a whole-brain searchlight analysis, this folder remains empty. Note that this is the only step in the whole processing stream where the user needs to intervene.

### 2.3. Labeling and classification

The PyMVPA BIDS-App uses functionality from PyMVPA to train a classifier on labeled images and validate the classifier using independent test data. In case of RSA, the (dis)similarity between labeled functional data points is computed and saved into a tab-separated values TSV. If enabled by the user through optional settings in the command-line interface of the app, each voxel/vertex is independently z-scored over time within each run (Pereira et al., 2009). This normalization is encouraged as heterogeneity in feature intensity can degrade the performance of some machine learning algorithms (Hanke et al., 2009), although the effect might be subtle (Misaki et al., 2010). Detrending of time-series (Mattay et al., 1996) can also be performed by the user at this level.

As part of the BIDS specification, experimental timing information is provided within TSV files that specify the onset and duration of events, located within each subject's directory. Boxcar functions constructed from the experimental timing are convolved with the hemodynamic response function (HRF) (Boynton et al., 1996) to generate predicted brain responses. A fixed-effects generalized linear model (GLM) implemented in PyMVPA uses the predicted response to estimate the event betas for each voxel/vertex; other predictors can also be included in the model with the user's intervention. An example with two conditions depicted in blue and red is shown in Figure 1. With ordinary least squares (OLS, default) as the fitting algorithm, betas can be estimated either per condition per run, or per trial per condition per run. The latter approach provides a higher number of training samples, while the former (depicted in Figure 1) better suits block designs and minimizes variance, although care must be taken as prolonging blocks can also introduce additional noise (Shan et al., 2014). A trade-off between the two should be made by the user by considering study design and data quality (Coutanche and Thompson-Schill, 2012; Abdulrahman and Henson, 2016; Zeithamova et al., 2017; Stehr et al., 2023). If fitting is performed in a least squares single (LSS) (Mumford et al., 2014) fashion, on the other hand, estimation will occur as follows: One single beta for the first trial and two sets (per condition per run) of betas for all the other trials will be estimated under one GLM. Subsequently, only the first trial's beta estimate will be saved. This process will be repeated for all the other trials, until they each have a corresponding estimate.

The beta series can then be subjected to a second z-scoring, which will be applied to beta estimates across all runs. This step is generally recommended for RSA analysis, and for classification would depend on the classifier type, as some SVM algorithms come with built-in across-run z-scoring (Cortes and Vapnik, 1995).

When running the pipeline in classification mode, beta estimates and labels are by default classified using a support vector machine (SVM), selected because of its reasonable performance with high-dimensional problems (Cortes and Vapnik, 1995). The classifier splits the data into a training fold that holds n-1 runs, and a second fold with a single run left for testing. This leave-one-run-out folding procedure can be modified through command-line flags if the user wishes to include more runs in the test split. For RSA analysis, the representational (dis)similarity between all pairs of beta estimates will be computed using the pairwise distance metric of “correlation”. Other metrics including “Euclidean” and “Mahalanobis” can also be used as specified through command-line arguments.

The PyMVPA BIDS-App returns an HTML report overview of the classification/RSA performance to maximize shareability between peers. The app also outputs spatial maps of classification accuracy in the format of NIfTI/GIfTI patterns that can be visualized in software packages such as FSLeyes or Neuropythy. If the analysis is restricted to an ROI, the HTML report will include a confusion matrix showing predictions vs. targets together with the overall classification accuracy. The classification patterns represent weights derived from the classifier for each voxel/vertex. A high weight indicates a relatively significant role in the decoding of experimental conditions, although care must be taken when interpreting such patterns as the meaning of weights changes from one classifier algorithm to another (Gaonkar et al., 2015). For searchlight classification, accuracy at each voxel/vertex represents the classifier's performance of the sphere/disc centered at that voxel/vertex.

## 3. Validation demonstrated with sample analyses

We demonstrate the PyMVPA BIDS-App by running it on two publicly available BIDS datasets. In our first analysis, we decode fMRI responses of a single subject to 8 categories of objects, obtained from the seminal (Haxby et al., 2001) MVPA study. This classification is implemented as an ROI-based approach restricted to the ventral temporal (VT) cortex. Within the same ROI, we will also illustrate the representational similarity structure of the object categories. The second analysis investigates the expression of two basic emotions of happiness and sadness during naturalistic viewing of the Forrest Gump movie (https://www.studyforrest.org/), implemented as whole-brain searchlight.

### 3.1. Object recognition in the ventral temporal cortex

In 2001, Haxby et al. (2001) introduced a new quantitative approach to evaluate the functional organization of the ventral visual pathway in humans. By investigating representational similarity of activation patterns, they showed that distinct representation of a stimulus category exists in the ventral temporal (VT) cortex not only exclusively in the region that responds maximally to that category as the traditional model suggested, but extending beyond even into areas that respond maximally to other categories. Included in this study were object categories of faces, houses, cats, bottles, scissors, shoes, chairs, and scrambled pictures. Here, we obtain a publicly available BIDS version of the dataset from the OpenNeuro database (ds000105) and perform 8-way classification on the aforementioned stimulus labels. Additionally, we obtain the representational similarity between the object categories. Both classification and RSA analyses are performed within a VT mask.

A total of 6 subjects are available on the OpenNeuro version of this dataset, however subjects 1–5 have lost orientation information in the T1-weighted image files due to a conversion issue (https://openfmri.org/dataset-orientation-issues/), and subject 6 lacks a high spatial resolution T1-weighted anatomical file. We, therefore, adopt anatomical data from another source available through the PyMVPA tutorial (http://data.pymvpa.org/datasets/tutorial_data). This lightweight version of the dataset includes only a single subject, Subject 1, which we use to complete this example analysis.

As the first preparatory step, the raw single-subject BIDS data is preprocessed using fMRIPrep (see Box 1 for details) and the functional data is registered to native space. All 12 functional scans for the subject are then concatenated into a single NIfTI file. Before the HRF modeling of the timeseries and performing classification/RSA, we restrict the analysis to a VT mask obtained from the PyMVPA tutorial database. This mask was constructed from a univariate GLM analysis using an 8-regressor model, with the first regressor being the contrast between stimulus blocks and rest, and the other seven being responses to each meaningful object category.

Box 1 **Invocations of both fMRIPrep and PyMVPA BIDS-App for the first sample analysis: object recognition in the VT cortex.**

*# fmriprep:*

docker run -ti --rm \

    -v [path to BIDS root]:/data \

    -v [path to FreeSurfer license.txt]:/opt/

    freesurfer/license.txt \

    nipreps/fmriprep:21.0.1 \

    /data /data/derivatives participant \

    --skip_bids_validation --participant-label 1

        --output-spaces func

*# preparation (concatenation):*

docker run -i --rm \

    -v [path to BIDS root]:/data \

    bids/pymvpa \

    /data /data/derivatives/pymvpa participant_prep\

    --participant_id 1 --task objectviewing

*# test (classification):*

docker run -i --rm \

    -v [path to BIDS root]:/data \

    bids/pymvpa \

    /data /data/derivatives/pymvpa participant_test\

    --participant_id 1 --task objectviewing --mask

    VT --bzscore \

    --conditions_to_classify bottle cat chair face

         house scissors scrambledpix shoe

*# test (rsa):*

docker run -i --rm \

    -v [path to BIDS root]:/data \

    bids/pymvpa \

    /data /data/derivatives/pymvpa participant_test\

    --participant_id 1 --task objectviewing --mask

    VT --bzscore --rsa

**Figure 2:**
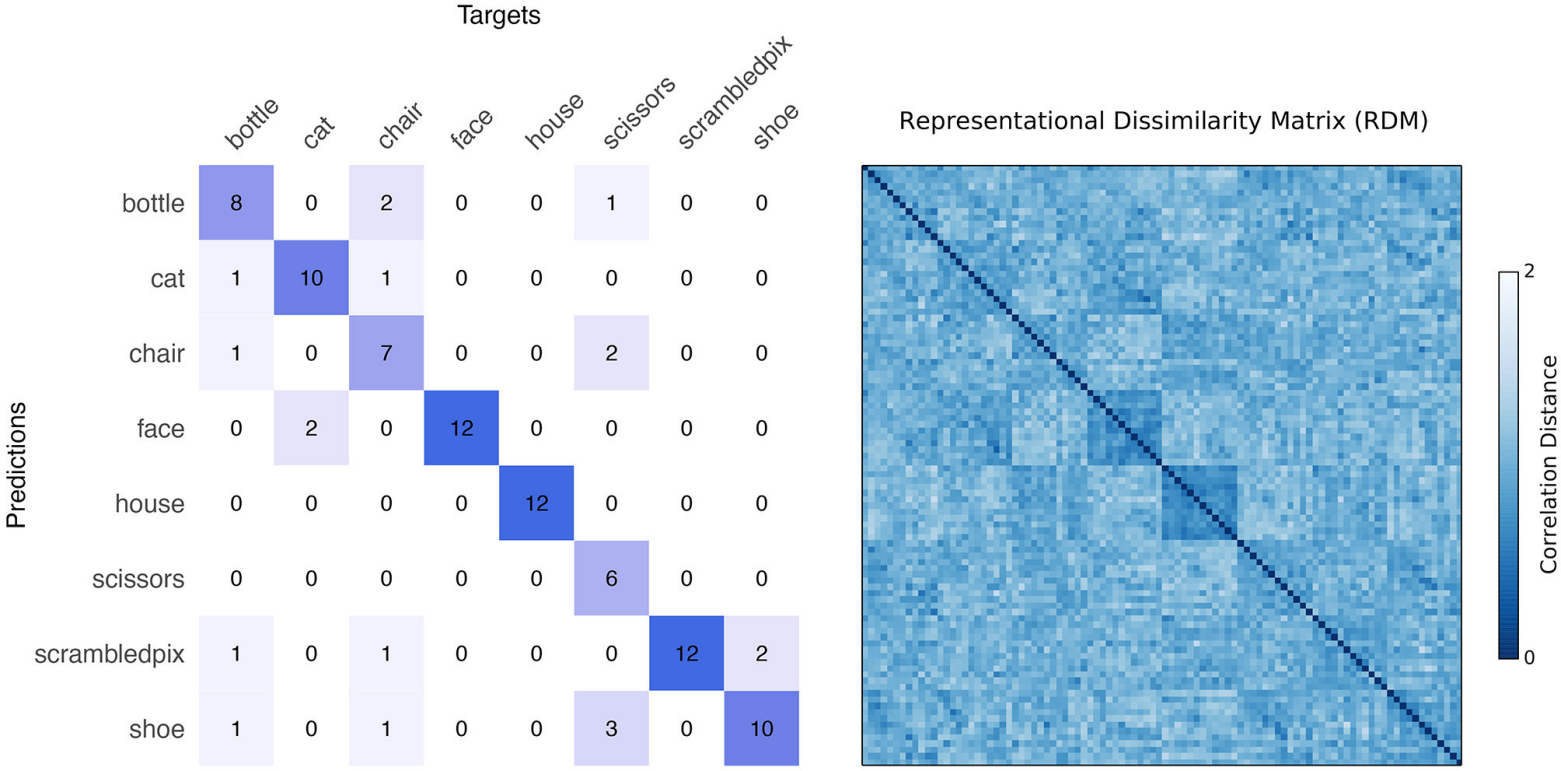
**Left**: Confusion matrix as returned by the PyMVPA BIDS-App for the classification of 8 object categories in the ventral temporal (VT) cortex. Rows and columns represent predicted and target categories, respectively. Each cell specifies the number of samples (out of 12) predicted as each of the 8 categories. An overall decoding performance of 80.21% (chance at 12.5%) is achieved, with all faces, houses, and scrambled pictures correctly classified as their own categories. Cross-validation is performed in a leave-one-run-out fashion. **Right**: Representational Dissimilarity Matrix (RDM) showing distance between samples of the 8 categories. For each category, 12 samples coming from 12 runs are used in the analysis. Distances are computed as correlation distance which range from 0 (most similar) to 2 (most dissimilar) and depict robust face and house categories, matching classification results.

The result of the 8-way leave-one-run-out classification is detailed in Figure 2, together with the representational dissimilarity matrix (RDM). Box 1 shows invocations of PyMVPA BIDS-App to generate these results. An overall classification accuracy of 80.21%—with chance being 12.5%—is achieved by estimating betas per condition per run, with z-scoring performed on the betas (features). Statistical significance is assessed using permutation testing in which the condition labels are randomized, resulting in classification performance as expected by chance. A null distribution built from 10,000 iterations reveals the likelihood of observing our true classification by chance to be *p* = 0.00009. Eliminating z-scoring results in somewhat weaker overall classification accuracy (68.75%), z-scoring on the timeseries only yields 75% accuracy, and z-scoring on both timeseries and beta estimates returns a robust 78.12%. Faces and houses are predicted with perfect precision, as expected due to their robust category-selective responses in the fusiform face area (FFA) (Kanwisher et al., 1997) and parahippocampal place area (PPA) (Epstein and Kanwisher, 1998), both of which were included in the VT mask. Categories of bottles, cats, chairs, scissors, scrambled, and shoes are predicted with 67%, 83%, 58%, 50%, 100%, and 83% accuracies, respectively.

The RDM in Figure 2 depicts correlation distances between all pairs of samples from the 8 categories. For each category, 12 samples are computed that correspond to the 12 runs in the experiment, forming a total of 8*12 rows (and columns) in the dissimilarity matrix. Blue face and house squares at the center of the RDM illustrate the robustness of these categories, which are classified nearly perfectly against the other categories. Interestingly, although the scrambled images were also distinct from the other categories, they do not show strong within category similarity, suggesting that the pattern representing one scrambled picture does not predict the pattern of activation for another scrambled picture.

Haxby et al. (2001)'s object recognition study employed a simplified blocked experimental design as was commonly used in many fMRI experiments. In the following analysis, we illustrate the flexibility and performance of the PyMVPA BIDS-App using a second dataset that involves diverse and complex sensory representations as conveyed during naturalistic viewing.

### 3.2. Searchlight decoding of emotions elicited by the movie Forrest Gump

The StudyForrest project (https://www.studyforrest.org/) seeks to take a major step toward a better understanding of how the brain works in real-life contexts by coupling neurophysiological measures with extensive, detailed annotations of perceptual, cognitive and emotional events (Labs et al., 2015) during naturalistic viewing. Here, we use this dataset to map brain regions with multivariate patterns that are associated with basic emotions.

The BIDS version of this dataset is available on the OpenNeuro database (ds000113). We specifically use the “movie” session of the data where 15 participants watched the full 2-h audio-visual version of the movie Forrest Gump split evenly across 8 runs. We preprocess the data using fMRIPrep with susceptibility distortion correction applied, as fieldmaps are missing in the movie session (see Box 2). Functional images are normalized to the standard MNI152NLin2009cAsym reference space.

Box 2 **Invocations of both fMRIPrep and PyMVPA BIDS-App for the second sample analysis: searchlight decoding of emotions.**

*# fmriprep:*

docker run -ti --rm \

    -v [path to BIDS root]:/data \

    -v [path to FreeSurfer license.txt]:/opt/

    freesurfer/license.txt \

    nipreps/fmriprep:21.0.1 \

    /data /data/derivatives participant

         --skip_bids_validation --participant-label

    01 02 03 04 05 06 09 10 14 15 16 17 18 19 20

         --ignore fieldmaps --use-syn-sdc

*# preparation (concatenation):*

docker run -i --rm \

    -v [path to BIDS root]:/data \

    bids/pymvpa \

    /data /data/derivatives/pymvpa participant_prep

         --participant_id 01 02 03 04 05 06 09 10 14

    15 16 17 18 19 20 --session movie --task movie

*# test (searchlight classification):*

docker run -i --rm \

    -v [path to BIDS root]:/data \

    bids/pymvpa \

    /data /data/derivatives/pymvpa participant_test

         --participant_id 01 02 03 04 05 06 09 10 14

     15 16 17 18 19 20 --session movie --task movie

         --searchlight 2.0 --tzscore --bzscore \

    --conditions_to_classify happiness sadness

An independent set of 9 observers (students at the Otto-von-Guericke-University in Magdeburg, Germany, all female) have annotated the complete movie outside the scanner. Each of these individuals have independently identified the onset and duration of episodes that portray emotions, semantic conflict, body contact, etc. Annotations of emotions include the basic attributes of arousal and valence, the identity of the movie character expressing the emotion, explicit emotion categories such as fear and love, and a variety of other features including face and audio. These observations are provided through inter-observer agreement (IOA) scores that reliably indicate the occurrence of an emotional attribute during each period of time (annotations). To achieve robustness in this analysis, emotional events are only included if the IOA scores reflect an emotional expression identified by least 5 of 9 observers. These events are used to generate predicted brain responses for a whole-brain searchlight analysis.

This analysis targets the basic emotions of happiness and sadness, as these two emotional expressions exist more frequently and more evenly distributed across the 8 runs of the movie as compared to other emotions. Among 14 basic and non-basic (e.g., hope, shame) emotions annotated, happiness and sadness together constitute 44% of all labels. We generate predicted brain responses using the timing and duration derived from the annotations on happiness and sadness, with betas derived from each run independently after z-scoring of timeseries. Beta estimates per condition (i.e., emotion) per run are z-scored then classified using a linear SVM in a leave-one-run-out cross-validation regime for each participant. Invocations of fMRIPrep and the PyMVPA BIDS-App for this analysis are shown in Box 2.

**Figure 3:**
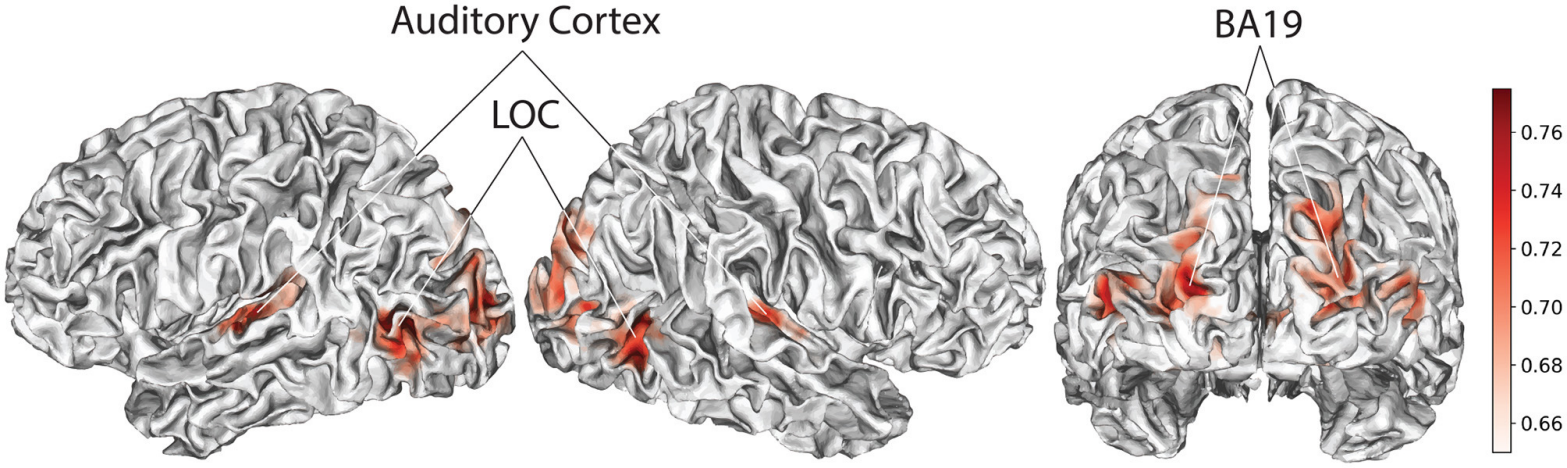
Searchlight results classifying happiness vs. sadness. Spatial maps denote accuracies with max at 77.5% (chance = 50%). Note that analyses are conducted in volumetric space, and then projected on the surface for visualization purposes using Neuropythy, an open-source library for surface-based analyses that complements the interface to volumetric images provided by the NiBabel library.

Figure 3 depicts the results of the whole-brain searchlight (spheres of radius 2) with classification accuracy for discriminating happiness vs. sadness, averaged across all subjects. Highest classification accuracies (max = 77.5%) are found in Brodmann area 19, the lateral occipital temporal cortex (LOC), and primary auditory cortex. We did not find above strong classification in the amygdala, which is traditionally known to be involved in processing aversive (LeDoux, 1996), and also pleasant (Janak and Tye, 2015) stimuli.

In naturalistic viewing, emotional expressions can take many forms and often depend on contextual elements such as the music played with a scene. To investigate what factors elicit the perception of the emotions of interest, we fit Generalized Estimating Equations (GEE) with logit links to the binarized IOA annotation scores, with happiness and sadness as dependent variables in two separate models, and include features of audio, face, gesture, and verbal communication as independent variables. Random effects are included to account for the variance coming from various characters in the movie. Note that we perform GEE and not Generalized Linear Mixed Models (GLMM) as specific characters are not of interest here. The results of our modeling on happiness reveal only a significant effect of audio ($\hat{\beta }=1.346,\chi^{2}=6.880,p=0.008$). The GEE model on sadness, on the other hand, shows significant effects of audio ($\hat{\beta }=-39.509,\chi^{2}=13753.98,p<2e-16$), face ($\hat{\beta }=1.292,\chi^{2}=14.68,p=0.00013$), and gesture ($\hat{\beta }=-1.308,\chi^{2}\;=\;5.39,p\;=\;0.0203$).

The high accuracy in the LOC and auditory cortex likely reflects the co-occurrence of emotional expression with salient facial and gestural (Weiner and Grill-Spector, 2013) and audio events, respectively. The LOC has also been reported in another study on emotions in the Forrest Gump movie (Lettieri et al., 2019), as one of the brain regions associated with ratings of the perceived intensity of six basic emotions including happiness and sadness. Distinct auditory patterns at a primary and not at higher ventral/dorsal auditory levels (Hickok and Poeppel, 2007), specifically, suggest the importance of early detection of basic emotions. Similar to the LOC, BA 19 has also been reported in studies of complex action observation (Molnar-Szakacs et al., 2006), which might be involved in conveying different emotions. Further analysis using more detailed annotations is however needed to draw conclusion on factors that drive basic emotions, for example by breaking gesture down to more specific action types with different limbs.

## 4. Discussion

The PyMVPA BIDS-App enables fMRI researchers to perform MVPA analyses including classification and RSA in a portable and highly reproducible environment. By integrating PyMVPA functionality into the BIDS standard, the app makes advanced MVPA research feasible for novice users, while offering more control to experts through an extensive set of command-line arguments, and through possible modifications to the open-source pipeline. One limitation of this work is its dependence of the pipeline on prior data preprocessing using the fMRIPrep BIDS-App, which provides the critical NIfTI/GIfTI images for use in volumetric/surface-based MVPA analysis. As a docker container, the PyMVPA BIDS-App makes the use of PyMVPA (together with all its dependencies) as easy as running a single terminal command, with no need to install software.

The PyMVPA BIDS-App runs in a variety of settings, including in volumetric or surface-based mode, and within ROIs or on the whole brain through searchlights. Furthermore, functionality that was previously non-existent in PyMVPA, including LSS model estimations, is now integrated into this BIDS-App version of PyMVPA. The app has already been deployed from the Docker Hub more than 1.4k times, and used in published work as well (e.g., O'Brien et al., 2022).

## 5. Materials and methods

### 5.1. fMRIPrep pre-processing of study 1

Results included in this manuscript come from preprocessing performed using *fMRIPrep* 20.1.1 (Esteban et al., 2018, 2023; RRID:SCR_016216), which is based on *Nipype* 1.5.0 (Gorgolewski et al., 2011; Esteban et al., 2021; RRID:SCR_002502).

#### 5.1.1. Anatomical data preprocessing

A total of 1 T1-weighted (T1w) images were found within the input BIDS dataset. The T1-weighted (T1w) image was corrected for intensity non-uniformity (INU) with N4BiasFieldCorrection (Tustison et al., 2010), distributed with ANTs 2.2.0 (Avants et al., 2008, RRID:SCR_004757) and used as T1w-reference throughout the workflow. The T1w-reference was then skull-stripped with a *Nipype* implementation of the antsBrainExtraction.sh workflow (from ANTs), using OASIS30ANTs as target template. Brain tissue segmentation of cerebrospinal fluid (CSF), white-matter (WM) and gray-matter (GM) was performed on the brain-extracted T1w using fast (FSL 5.0.9, RRID:SCR_002823, Zhang et al., 2001). Brain surfaces were reconstructed using recon-all (FreeSurfer 6.0.1, RRID:SCR_001847, Dale et al., 1999), and the brain mask estimated previously was refined with a custom variation of the method to reconcile ANTs-derived and FreeSurfer-derived segmentations of the cortical gray-matter of Mindboggle (RRID:SCR_002438, Klein et al., 2017). Volume-based spatial normalization to one standard space (MNI152NLin2009cAsym) was performed through non-linear registration with antsRegistration (ANTs 2.2.0), using brain-extracted versions of both T1w reference and the T1w template. The following template was selected for spatial normalization: *ICBM 152 Nonlinear Asymmetrical template version 2009c* (Fonov et al., 2009, RRID:SCR_008796; TemplateFlow ID: MNI152NLin2009cAsym).

#### 5.1.2. Functional data preprocessing

For each of the 12 BOLD runs found per subject (across all tasks and sessions), the following preprocessing was performed. First, a reference volume and its skull-stripped version were generated using a custom methodology of *fMRIPrep*. Head-motion parameters with respect to the BOLD reference (transformation matrices, and six corresponding rotation and translation parameters) are estimated before any spatiotemporal filtering using mcflirt (FSL 5.0.9, Jenkinson et al., 2002). Susceptibility distortion correction (SDC) was omitted. The BOLD reference was then co-registered to the T1w reference using bbregister (FreeSurfer) which implements boundary-based registration (Greve and Fischl, 2009). Co-registration was configured with six degrees of freedom. The BOLD time-series (including slice-timing correction when applied) were resampled onto their original, native space by applying the transforms to correct for head-motion. These resampled BOLD time-series will be referred to as *preprocessed BOLD in original space*, or just *preprocessed BOLD*. Several confounding time-series were calculated based on the *preprocessed BOLD*: framewise displacement (FD), DVARS and three region-wise global signals. FD was computed using two formulations following Power (absolute sum of relative motions, Power et al., 2014) and Jenkinson (relative root mean square displacement between affines, Jenkinson et al., 2002). FD and DVARS are calculated for each functional run, both using their implementations in *Nipype* (following the definitions by Power et al., 2014). The three global signals are extracted within the CSF, the WM, and the whole-brain masks. Additionally, a set of physiological regressors were extracted to allow for component-based noise correction (*CompCor*, Behzadi et al., 2007). Principal components are estimated after high-pass filtering the *preprocessed BOLD* time-series (using a discrete cosine filter with 128s cut-off) for the two *CompCor* variants: temporal (tCompCor) and anatomical (aCompCor). tCompCor components are then calculated from the top 5% variable voxels within a mask covering the subcortical regions. This subcortical mask is obtained by heavily eroding the brain mask, which ensures it does not include cortical GM regions. For aCompCor, components are calculated within the intersection of the aforementioned mask and the union of CSF and WM masks calculated in T1w space, after their projection to the native space of each functional run (using the inverse BOLD-to-T1w transformation). Components are also calculated separately within the WM and CSF masks. For each CompCor decomposition, the *k* components with the largest singular values are retained, such that the retained components' time series are sufficient to explain 50 percent of variance across the nuisance mask (CSF, WM, combined, or temporal). The remaining components are dropped from consideration. The head-motion estimates calculated in the correction step were also placed within the corresponding confounds file. The confound time series derived from head motion estimates and global signals were expanded with the inclusion of temporal derivatives and quadratic terms for each (Satterthwaite et al., 2013). Frames that exceeded a threshold of 0.5 mm FD or 1.5 standardized DVARS were annotated as motion outliers. All resamplings can be performed with *a single interpolation step* by composing all the pertinent transformations (i.e., head-motion transform matrices, susceptibility distortion correction when available, and co-registrations to anatomical and output spaces). Gridded (volumetric) resamplings were performed using antsApplyTransforms (ANTs), configured with Lanczos interpolation to minimize the smoothing effects of other kernels (Lanczos, 1964). Non-gridded (surface) resamplings were performed using mri_vol2surf (FreeSurfer).

Many internal operations of *fMRIPrep* use *Nilearn* 0.6.2 (Abraham et al., 2014, RRID:SCR_001362), mostly within the functional processing workflow. For more details of the pipeline, see the section corresponding to workflows in *fMRIPrep*'s documentation.

### 5.2. fMRIPrep pre-processing of study 2

Results included in this manuscript come from preprocessing performed using *fMRIPrep* 20.1.1 (Esteban et al., 2018, 2023; RRID:SCR_016216), which is based on *Nipype* 1.5.0 (Gorgolewski et al., 2011; Esteban et al., 2021; RRID:SCR_002502).

#### 5.2.1. Anatomical data preprocessing

A total of 1 T1-weighted (T1w) images were found within the input BIDS dataset. The T1-weighted (T1w) image was corrected for intensity non-uniformity (INU) with N4BiasFieldCorrection (Tustison et al., 2010), distributed with ANTs 2.2.0 (Avants et al., 2008, RRID:SCR_004757), and used as T1w-reference throughout the workflow. The T1w-reference was then skull-stripped with a *Nipype* implementation of the antsBrainExtraction.sh workflow (from ANTs), using OASIS30ANTs as target template. Brain tissue segmentation of cerebrospinal fluid (CSF), white-matter (WM) and gray-matter (GM) was performed on the brain-extracted T1w using fast (FSL 5.0.9, RRID:SCR_002823, Zhang et al., 2001). Brain surfaces were reconstructed using recon-all (FreeSurfer 6.0.1, RRID:SCR_001847, Dale et al., 1999), and the brain mask estimated previously was refined with a custom variation of the method to reconcile ANTs-derived and FreeSurfer-derived segmentations of the cortical gray-matter of Mindboggle (RRID:SCR_002438, Klein et al., 2017). Volume-based spatial normalization to one standard space (MNI152NLin2009cAsym) was performed through non-linear registration with antsRegistration (ANTs 2.2.0), using brain-extracted versions of both T1w reference and the T1w template. The following template was selected for spatial normalization: *ICBM 152 Nonlinear Asymmetrical template version 2009c* (Fonov et al., 2009, RRID:SCR_008796; TemplateFlow ID: MNI152NLin2009cAsym).

#### 5.2.2. Functional data preprocessing

For each of the 8 BOLD runs found per subject (across all tasks and sessions), the following preprocessing was performed. First, a reference volume and its skull-stripped version were generated using a custom methodology of *fMRIPrep*. Head-motion parameters with respect to the BOLD reference (transformation matrices, and six corresponding rotation and translation parameters) are estimated before any spatiotemporal filtering using mcflirt (FSL 5.0.9, Jenkinson et al., 2002). BOLD runs were slice-time corrected using 3dTshift from AFNI 20160207 (Cox and Hyde, 1997, RRID:SCR_005927). A deformation field to correct for susceptibility distortions was estimated based on *fMRIPrep*'s *fieldmap-less* approach. The deformation field is that resulting from co-registering the BOLD reference to the same-subject T1w-reference with its intensity inverted (Huntenburg, 2014; Wang et al., 2017). Registration is performed with antsRegistration (ANTs 2.2.0), and the process regularized by constraining deformation to be non-zero only along the phase-encoding direction, and modulated with an average fieldmap template (Treiber et al., 2016). Based on the estimated susceptibility distortion, a corrected EPI (echo-planar imaging) reference was calculated for a more accurate co-registration with the anatomical reference. The BOLD reference was then co-registered to the T1w reference using bbregister (FreeSurfer) which implements boundary-based registration (Greve and Fischl, 2009). Co-registration was configured with six degrees of freedom. The BOLD time-series (including slice-timing correction when applied) were resampled onto their original, native space by applying a single, composite transform to correct for head-motion and susceptibility distortions. These resampled BOLD time-series will be referred to as *preprocessed BOLD in original space*, or just *preprocessed BOLD*. The BOLD time-series were resampled into standard space, generating a *preprocessed BOLD run in MNI152NLin2009cAsym space*. First, a reference volume and its skull-stripped version were generated using a custom methodology of *fMRIPrep*. Several confounding time-series were calculated based on the *preprocessed BOLD*: framewise displacement (FD), DVARS and three region-wise global signals. FD was computed using two formulations following Power (absolute sum of relative motions, Power et al., 2014) and Jenkinson (relative root mean square displacement between affines, Jenkinson et al., 2002). FD and DVARS are calculated for each functional run, both using their implementations in *Nipype* (following the definitions by Power et al., 2014). The three global signals are extracted within the CSF, the WM, and the whole-brain masks. Additionally, a set of physiological regressors were extracted to allow for component-based noise correction (*CompCor*, Behzadi et al., 2007). Principal components are estimated after high-pass filtering the *preprocessed BOLD* time-series (using a discrete cosine filter with 128s cut-off) for the two *CompCor* variants: temporal (tCompCor) and anatomical (aCompCor). tCompCor components are then calculated from the top 5% variable voxels within a mask covering the subcortical regions. This subcortical mask is obtained by heavily eroding the brain mask, which ensures it does not include cortical GM regions. For aCompCor, components are calculated within the intersection of the aforementioned mask and the union of CSF and WM masks calculated in T1w space, after their projection to the native space of each functional run (using the inverse BOLD-to-T1w transformation). Components are also calculated separately within the WM and CSF masks. For each CompCor decomposition, the *k* components with the largest singular values are retained, such that the retained components' time series are sufficient to explain 50 percent of variance across the nuisance mask (CSF, WM, combined, or temporal). The remaining components are dropped from consideration. The head-motion estimates calculated in the correction step were also placed within the corresponding confounds file. The confound time series derived from head motion estimates and global signals were expanded with the inclusion of temporal derivatives and quadratic terms for each (Satterthwaite et al., 2013). Frames that exceeded a threshold of 0.5 mm FD or 1.5 standardized DVARS were annotated as motion outliers. All resamplings can be performed with *a single interpolation step* by composing all the pertinent transformations (i.e., head-motion transform matrices, susceptibility distortion correction when available, and co-registrations to anatomical and output spaces). Gridded (volumetric) resamplings were performed using antsApplyTransforms (ANTs), configured with Lanczos interpolation to minimize the smoothing effects of other kernels (Lanczos, 1964). Non-gridded (surface) resamplings were performed using mri_vol2surf (FreeSurfer).

Many internal operations of *fMRIPrep* use *Nilearn* 0.6.2 (Abraham et al., 2014, RRID:SCR_001362), mostly within the functional processing workflow. For more details of the pipeline, see the section corresponding to workflows in *fMRIPrep*'s documentation.

## Data availability statement

The original contributions presented in the study are included in the article/supplementary material, further inquiries can be directed to the corresponding author.

## Ethics statement

This is a secondary analysis of anonymized data available through open access data sharing repositories. As cited in the original publications, the primary research study procedures were approved by the appropriate local institutional ethics committees in accordance with relevant institutional requirements and the Declaration of Helsinki.

## Author contributions

ST contributed with conceptualization, data curation, formal analysis, investigation, methodology, software, validation, visualization, and writing (original draft, review, and editing). NV contributed with conceptualization, formal analysis, investigation, methodology, validation, and writing (review and editing). VS contributed with methodology, software, and writing (review and editing). YH contributed with formal analysis, methodology, software, validation, and writing (review and editing). EG contributed with formal analysis, investigation, methodology, validation, visualization, and writing (original draft, review, and editing). All authors contributed to the article and approved the submitted version.
